# Individual differences in emotional intelligence skills of people with visual impairment and loneliness amid the COVID-19 pandemic

**DOI:** 10.1177/02646196211013860

**Published:** 2023-01

**Authors:** Hyung Nam Kim, Sam Jotham Sutharson

**Affiliations:** North Carolina Agricultural and Technical State University, USA

**Keywords:** COVID-19, emotional well-being, pandemic, social distancing, visual impairment

## Abstract

In response to the novel coronavirus (COVID-19) pandemic, public health interventions such as social distancing and stay-at-home orders have widely been implemented, which is anticipated to contribute to reducing the spread of COVID-19. On the contrary, there is a concern that the public health interventions may increase the level of loneliness. Loneliness and social isolation are public health risks, closely associated with serious medical conditions. As COVID-19 is new to us today, little is known about emotional well-being among people with visual impairment during the COVID-19 pandemic. To address the knowledge gap, this study conducted phone interviews with a convenience sample of 31 people with visual impairment. The interview incorporated the University of California, Los Angeles (UCLA) Loneliness Scale (version 3) and the trait meta-mood scale (TMMS) to measure loneliness and emotional intelligence skills, respectively. This study found that people with visual impairment were vulnerable to the feeling of loneliness during the COVID-19 pandemic and showed individual differences in emotional intelligence skills by different degrees of loneliness. Researchers and health professionals should consider offering adequate coping strategies to those with visual impairment amid the COVID-19 pandemic.

## Introduction

On 11 March 2020, the World Health Organization (WHO, 2020) declared the novel coronavirus (COVID-19) outbreak a global pandemic as over 118,000 cases were found in 114 countries and over 4000 people have lost their lives. The WHO Director-General noted at a news briefing that the WHO is “deeply concerned both the alarming levels of spread and severity and by the alarming levels of inaction” (WHO, 2020).” As of 8 October 2020, there were 7,528,313 cases and 211,132 deaths associated with COVID-19 in the United States (Centers for Disease Control and Prevention, 2020a). In response to the COVID-19 pandemic, stay-at-home orders, curfews, quarantines, and similar public health interventions were enforced. Only essential businesses were allowed to remain open, but the others (e.g., schools, daycares, colleges, and universities) were forced to close. Czeisler et al. (2020) surveyed adults in New York, Los Angeles, and broadly across in the United States and reported on 12 June 2020 that 79.5% of respondents supported the stay-at-home orders and nonessential business closures.

While safety measures can help to reduce the spread of COVID-19, contributing to physical health (Centers for Disease Control and Prevention, 2020b), a concern about mental health has ironically increased (Brooks et al., 2020; Holmes et al., 2020; Mahase, 2020). Among various mental health issues, a lot of attention has been given to loneliness (Berg-Weger & Morley, 2020; Roy, Jain, Golamari, Vunnam, & Sahu, 2020). When an individual has come into contact with the COVID-19 virus, more severe social isolation and quarantine restrictions would be imposed, for example, he or she is advised to completely self-isolate for at least 14 days. As people would experience such a prolonged separation from other people, they are likely to feel lonely, which is anticipated to further increase over time (Killgore, Cloonan, Taylor, & Dailey, 2020). A recent study in Germany reported that mental health worsened immediately after the onset of lockdown (Armbruster & Klotzbücher, 2020). A survey study with community-dwelling older adults in the Netherlands (van Tilburg, Steinmetz, Stolte, van der Roest, & de Vries, 2020) was conducted 2 months after the public health interventions were implemented and found that the loneliness of older adults has significantly increased. Another research team (Kotwal et al., 2020) conducted phone interviews with older adults living in the San Francisco Bay area in the United States during the COVID-19 shelter-in-orders and found that loneliness persisted or even worsened.

To date, not much has been known about the impact of COVID-19 on people with visual impairment. In May 2020, researchers in the United Kingdom (Ting, Krause, Said, & Dua, 2020) conducted a quick questionnaire (e.g., 27 items with 3-point Likert- type scale) to explore the perspective of people with eye diseases on the impact of COVID-19 pandemic lockdown. They found that people with moderate or severe visual impairments in the United Kingdom were three times more likely to report increased loneliness than their peers with mild or no visual impairments. The American Foundation for the Blind (AFB, 2020) also conducted a quick survey between 3 and 13 April 2020 (i.e., early stages of the US responses to COVID-19) for adults who have low vision or blind (female, 63.4%; Black or African American, 7%; age 54 and younger, 57.5%; living alone, 33.6%). The survey respondents shared various needs and concerns about the use of technology, transportation, healthcare, education, employment, and social experiences. For example, 68% of survey respondents had fears that they would not be able to get themselves or their loved ones to COVID-19 test sites or healthcare providers if they were to get sick amid the pandemic; 59% of respondents believed that their underlying conditions made them feel more vulnerable to COVID-19; 56% had fears related to their ability of practicing social distancing, asking for physical assistance, and using a sense of touch (e.g., using tactile sign language) as COVID-19 is a highly contagious pathogenic viral infection; and 21% were concerned about the telehealth platform, not accessible to people with visual impairment. Unfortunately, there is still lack of understanding about specific factors leading to mental health challenges during the COVID-19 pandemic (Holmes et al., 2020) especially among people with visual impairment. This study aims to advance knowledge of emotional well-being issues of those with visual impairment.

## Methods

### Participants

A convenience sampling method helped to invite 31 people with visual impairment across North Carolina in the United States. Research participants should speak English, be 18 years old or older, and have visual impairment (i.e., visual acuity level worse than 20/70 (WHO, 2008). Table 1 shows the participants’ characteristics.

**Table 1.** Characteristics of the participants.

Participants	*n* = 31
Visual acuity	
Between 20/70 and 20/200	2
Between 20/200 and 20/400	6
Between 20/400 and 20/1200	2
Less than 20/1200, but has light perception	19
No light perception at all	2
Duration of visual impairment (years)	29.30 ± 22.55
Onset of visual impairment (years)^a^	
Early onset (*n* = 9)	6.56 ± 2.60
Late onset (*n* = 22)	38.60 ± 20.28
Age (years)	62.84 ± 15.47
Gender	
Male	15
Female	16
Race/Ethnicity	
Black/African American	10
White	20
Native American	1
Marital status	
Married	15
Not Married	7
Widow/Widower	5
Divorced	3
Declined to answer	1
Education	
High school or equivalent	9
Associates	7
Bachelors	10
Masters	4
Doctorate	1
Occupation	
Full-time	3
Part-time	3
Unemployed	2
Retired	22
Declined to answer	1
Household income	
⩽US$25,999	7
US$26,000–US$51,999	5
US$52,000–US$74,999	2
⩾US$75,000	5
Declined to answer	12
Head of household	
Living alone	8
With family, relatives, friends, or combination of them	23
Diagnosed with health conditions	14
Participation in physical exercise	21

a Participants with early-onset visual impairment had lost their sight before they reached 11 years of age, while those with late-onset visual impairment had lost their sight after the age of 16 years (Voss, Gougoux, & Guillemot, 2004).

### Materials

#### University of California, Los Angeles Loneliness Scale (version 3)

Loneliness was measured with the University of California, Los Angeles (UCLA) Loneliness Scale, version 3 (α ranging from .89 to .94) that is a 20-item self-report measure designed to assess one’s subjective feelings of loneliness (Russell, 1996; Russell, Peplau, & Cutrona, 1980). Participants should rate each item on a 4-point Likert-type scale ranging from 1 = *never* to 4 = *often*. The possible range of scores is between 20 and 80, and higher scores indicate greater loneliness. A score ⩾65 indicates a severe high degree of loneliness, a score ⩾50 indicates a moderately high degree, a score ⩾35 indicates a moderate degree, and a score <35 indicates a low degree (Kusaslan Avci, 2018; Perry, 1990).

#### Emotional intelligence via trait meta-mood scale

Emotional intelligence was measured with the trait meta-mood scale (TMMS; Salovey, Mayer, Goldman, Turvey, & Palfai, 1995). The TMMS is a 30-item self-report measure designed to assess the attention to the feelings (Cronbach’s α = .86), the clarity of the emotional experiences (Cronbach’s α = .88), and the repair of negative emotions (Cronbach’s α = .82) (Salovey et al., 1995). The Attention scale includes 13 items (e.g., “I pay a lot of attention to how I feel,” and “I don’t think it’s worth paying attention to your emotions or moods”). The Clarity scale includes 11 items (e.g., “I am usually very clear about my feelings” and “I can’t make sense out of my feelings”). The Repair scale includes six items (e.g., “When I become upset, I remind myself of all the pleasures in life” and “I try to have good thoughts no matter how bad I feel”). Participants should complete the TMMS using a 5-point Likert-type scale, with options ranging from 1 = *strongly disagree* to 5 = *strongly agree*.

### Procedures

Participants were recruited with supports from community organizations that provide services for people with visual impairment (e.g., community centers, a library for the blind) in North Carolina, USA. Approval for this study was obtained from the Institutional Review Board (IRB). The research team administered the questionnaires via phone between May and August 2020 during the COVID-19 pandemic.

### Data analysis

The data were analyzed using the descriptive statistics, the Cronbach’s α for internal consistency, and the Kruskal–Wallis test with the post hoc Mann–Whitney test. Correlation was examined with Spearman’s rho. Statistical analyses were performed using the IBM SPSS Statistics for Macintosh, version 24 (IBM Corp., 2016).

## Results

### Emotional intelligence and loneliness

The TMMS for emotional intelligence showed adequate internal consistency (Cronbach’s α = .79), and the overall emotional intelligence score was 3.75 ± 1.40, which can be broken down into three groups: attention (3.51 ± 1.43), clarity (3.70 ± 1.43), and repair (4.33 ± 1.13). The UCLA scale for loneliness showed adequate internal consistency (Cronbach’s α = .93), and the overall loneliness score was 39.19 ± 14.04. The following items showed higher loneliness scores that are above the upper quartile (i.e., the highest 25% of the data): “My interests and ideas are not shared by those around me,” “I feel left out,” “I feel isolated from others,” and “People are around me but not with me.” All participants experienced loneliness, ranging from low to severe, that is, 14 participants had a low degree of loneliness (26.86 ± 3.84), 9 had a moderate degree (42.20 ± 6.01), 5 had a moderately high degree (56.20 ± 4.09), and 2 had a severely high degree (68.00 ± 1.41). For the data analysis purpose, participants with a severely high degree and those with a moderately high degree were combined in a single group, named a group with a high degree of loneliness. Thus, participants were sorted into three groups: one with a low degree of loneliness, one with a moderate degree, and one with a high degree. The Kruskal–Wallis test indicated that the three groups had significantly different loneliness scores, *H*(2) = 25.25, *p* < .001. The post hoc Mann–Whitney tests with Bonferroni correction were performed to follow up on the significant findings. All the three groups had significantly different loneliness scores: a group with low loneliness versus a group with moderate loneliness (*U* = 2.50, *z* = −3.96, *p* < .001, *r* = −.81), a group with moderate loneliness versus a group with high loneliness (*U* = 0, *z* = −3.43, *p<* .001, *r* = −.83), and a group with high loneliness versus a group with low loneliness (*U* = 0, *z* = −3.66, *p* < .001, *r* = −.80).

### Relationship between emotional intelligence and loneliness

The following two abilities of emotional intelligence – *the ability of attention to the feelings* and *the ability of clarity of the emotional experiences* – showed no significant relationship with loneliness. Yet, there was a significant negative correlation between loneliness and repair ability of negative emotions, *r* = −.68, *p* < .01. The Kruskal–Wallis test indicated that the three groups with different loneliness levels (low, moderate, and high) had significantly different repair ability scores, *H*(2) = 11.38, *p* = .003. The post hoc Mann–Whitney test with Bonferroni correction was performed to follow up on the significant findings. The following groups had significantly different repair ability scores: a low loneliness group (4.69 ± 0.44) versus a moderate loneliness group (4.17 ± 0.56) (*U* = 29.50, *z* = −2.43, *p* = .015, *r* = −.5) and a low loneliness group (4.69 ± 0.44) versus a high loneliness group (3.83 ± 0.52) (*U* = 9.00, *z* = −3.076, *p* = .002, *r* = −.67). The results suggest that a higher level of loneliness is likely to be observed in people with a lower level of repair ability.

### Relationship between emotional intelligence and loneliness among subgroups of participants sorted by sociodemographic factors

After grouping participants by sociodemographic factors, we examined the relationship between emotional intelligence and loneliness. Although there was no significant correlation between clarity and loneliness, significant correlations were found between repair and loneliness, and between attention and loneliness. Among significant relationships, all were negative relationships except one case, that is, a positive relationship was found between attention and loneliness among participants who did not exercise. The results suggest that the repair and attention abilities of emotional intelligence are closely related to loneliness management across the participants with the following sociodemographic backgrounds (see Table 2), and those with no exercise are likely to experience that the more they pay attention to emotions, the more they feel lonely.

**Table 2.** Correlation between loneliness and emotional intelligence among participants grouped by sociodemographic factors.

Group	Correlation between repair and loneliness	Correlation between attention and loneliness
European Americans	*r* = −.696, *p* < .01	
Older adults	*r* = −.696, *p* < .01	
People with blindness	*r* = −.651, *p* < .01	
Early onset	*r* = −.694, *p<.05*	
Late onset	*r* = −.666, *p* < .01	
Male	*r* = −.754, *p* < .01	
Female	*r* = −.615, *p<.05*	
People with college degree	*r* = −.739, *p<.05*	
People with associate degree		*r* = −.829, *p<.05*
Household income, US$25,000–US$75,000	*r* = −.894, *p<.05*	*r* = −.900, *p<.05*
Living with family, relatives, friends, or combination of them	*r* = −.676, *p* < .01	
No significant health problems	*r* = −.687, *p* < .01	
Exercise	*r* = −.716, *p* < .01	*r* = −.563, *p* < .01
No exercise		*r* = .735, *p<.05*

As shown in Table 3, Mann–Whitney test for between-groups with Bonferroni correction indicated that significantly different mean scores of loneliness were found among male and female groups, while significantly different mean scores of repair and attention were found among African American and European American groups.

**Table 3.** Mean differences of loneliness and emotional intelligence among participants grouped by sociodemographic factors.

Variables		Groups	Mean ± *SD*	Mann–Whitney with Bonferroni correction
Loneliness		Male	34.47 ± 14.25	*U* = 63.5, *z* = −2.24, *p=* .025, *r* = −.40
		Female	43.63 ± 12.71	
Emotional intelligence	Repair	African American	3.92 ± 0.60	*U* = 44.5, *z* = −2.48, *p=* .01, *r* = −.45
		European American	4.56 ± 0.50	
	Attention	African American	3.18 ± 0.46	*U* = 41.5, *z* = −2.58, *p=* .01, *r* = −.47
		European American	3.70 ± 0.58	

*SD*: standard deviation.

### Various emotional experiences of participants with visual impairment during the COVID-19 pandemic

In addition to completing the UCLA loneliness questionnaire, participants briefly described emotions that they experienced during the COVID-19 pandemic. As shown in Table 4, they felt isolated, left out, being ignored, pessimistic, superficial relationship, unhappy, upset, and uncomfortable. An inter-rater reliability analysis using Cohen’s kappa statistic was performed to determine consistency between two raters. There was substantial agreement among the raters as the inter-rater reliability was found to be κ = 0.88 (95% confidence interval [CI]: 0.649 to 1.119).

**Table 4.** Emotions of those with visual impairment during the COVID-19 pandemic.

Emotions	Participants’ comments
Feeling isolated	• *I have family and friends but still feel isolated due to COVID-19.* • *I have close relationships with my immediate family only, but not other people.* • *I [the elderly] have adult children but not living together, so I do not feel close to family either.* • *I do not feel part of a group of friends because COVID-19 prohibits gathering.* • *COVID-19 and social distancing make me feel isolated.*
Feeling left out	• *I feel left out when I encounter poor accessibility to communication technologies*
Feeling of being ignored	• *It feels like no one listens to me and cares about my feelings.* • *People do not know my well-being.*
Feeling lonely	• *I become lonely due to the COVID19 pandemic.* • *I become much lonelier since the COVID19 pandemic.*
Feeling pessimistic	• *I become more pessimistic because people have ignored my concern about this pandemic.* • *COVID-19 has changed my outlook to be pessimistic.*
Feeling superficial relationship	• *I feel like I have many acquaintances but not real friends in my life.* • *I helped people but people did not help me. It is like a one-way street instead of a two-way street. My relationship is superficial.* • *I have people who think they understand me, but I am unsure about it.* • *People around me but not with me due to COVID-19.*
Feeling unhappy	• *I am unhappy because I am losing my independence due to the COVID-19 pandemic*
Feeling upset	• *I feel upset because of COVID-19.*
Feeling uncomfortable	• *I feel uncomfortable because COVID-19 makes me go out with someone always.*

While completing the TMMS emotional intelligence questionnaire for measuring attention, clarity, and repair, participants also briefly discussed their strategies of managing the emotions. As shown in Table 5, those strategies were then grouped by management styles – that is, avoid, alter, accept, and adapt. An inter-rater reliability analysis using Cohen’s kappa statistic was performed to determine consistency between two raters. There was substantial agreement among the raters as the inter-rater reliability was found to be κ = 0.69 (95% CI: 0.323 to 1.062). The participants tended to avoid the situations that might lead to emotional challenges; however, if they could not keep themselves from the problematic sources, they tried to alter the situations. When the alteration was not successful, they would simply accept the situations the way they were. Yet, if they felt they could not accept the situations, they tried to change their own standards or expectations (i.e., adapting) to deal with what they encountered.

**Table 5.** Emotional intelligence and management types.

Emotional intelligence	Participants’ comments	Management types
Attention	• *I try not to pay attention to emotions because it would cause many problems in life.*	Avoid
	• *I write my thoughts (or talk about them) in order to pay attention to emotions.* • *I think out loud and express emotions to others.* • *I pay attention to emotions only if it is a negative emotion.*	Alter
	• *I found myself giving in to emotions when depressed, so I talked with family and felt better after talking it out.*	Accept
Clarity	• *I have the ability to identify and screen out people with superficial relationships.*	Avoid
	• *I rely on my intuition to understand my emotions, but often cannot understand it exactly.* • *I would not need to make effort to clarify my emotions because I am always happy.*	Accept
Repair	• *I choose to live in the present instead of focusing on things that happened in the past.*	Avoid
	• *I make phone calls or meet friends by keeping social distancing in order to manage my loneliness, instead of staying at home alone.* • *I feel a lack of companionship, but I often meet my family members.* • *I attend a social support group and meet people.* • *I participate in online social networking.*	Alter
	• *I believe that emotions are one that cannot be controlled at all.* • *You cannot predict your emotions. You just experience them as they come and go.*	Accept
	• *I lift myself up when being upset and angry.* • *I remind myself of all the pleasures in my life.* • *Due to my faith in God, I feel stronger in managing my emotions.* • *I feel lonely, but I believe that it will get better sooner or later after the COVID-19 pandemic is over.* • *It would be okay encountering people with different interests and ideas because it is an opportunity for me to learn something new.* • *I pretend to be an outgoing person (i.e., pseudo-extravert).* • *I ask myself not to share any feelings with anyone as I would like to hide my depression.*	Adapt

## Discussion

This study explored the degree to which a sample of people with visual impairment (age 62.84 ± 15.47) experienced a feeling of loneliness amid the COVID-19 pandemic. None of them reported non-loneliness as their loneliness levels ranged from low to severe high, and the overall mean of loneliness scores (39.19 ± 14.04) was higher than that (31.51 ± 6.92) of sighted people (age 65 and over) in the study by Russell (1996), one of the most widely cited loneliness studies. Therefore, it could hypothetically be argued that during the COVID-19 pandemic, higher loneliness is likely to be observed in adults with visual impairment compared to their sighted peers. As COVID-19 is a new disease caused by a novel coronavirus today, only a few reports (Grey et al., 2020; Killgore et al., 2020; Killgore, Cloonan, Taylor, Miller, & Dailey, 2020; Luchetti et al., 2020) have been published with regard to loneliness of people amid the COVID-19 pandemic, yet most of them adopted a short version (3 items) of the original UCLA loneliness scale (a full list of 20 items) and, furthermore, merely focused on general populations. This study used the full version such that it can provide more in-depth understandings of loneliness, especially among those with visual impairment.

The overall mean loneliness score in this study was also higher than that (38.4 ± 13.5) of prior research by Morahan-Martin and Schumacher (2003) in which loneliness in undergraduate students was measured using the UCLA Loneliness Scale (a full version) and found to be associated with increased Internet use. Lonely undergraduate students were more likely to use the Internet to obtain emotional support and regulate negative moods, compared to their peer students who were not lonely. The Internet might be considered as an alternative channel to keep interactions with people with visual impairment amid the COVID-19 pandemic; however, as shown in the study by Morahan-Martin and Schumacher (2003), the Internet use can be effective only for a short term and cannot be an ideal solution to cope with loneliness in the long term.

Loneliness of the participants with visual impairment was not significantly correlated with the ability of attention to the feelings and the ability of clarity of the emotional experiences; however, it was significantly correlated with the ability of repair of negative emotions. Those with a higher level of repair ability are more likely to successfully manage their emotions, for example, terminate negative mood states, restructure the situation, prolong pleasant emotions, calm down, and/or use distraction strategies (Fernandez-Berrocal & Extremera, 2008). Loneliness is viewed as a subjective experience and is observed in people who experience a discrepancy between the current and the desired levels of interpersonal relations (Perlman & Peplau, 1981), which is likely affected by social isolation and one’s perceived loneliness. Social isolation refers to the objectively quantified deficit in one’s social relationships (de Jong Gierveld, Van Tilburg, & Dykstra, 2006). Due to the COVID-19 pandemic, people with visual impairment are less likely to maintain an in-person social interaction with other people, probably leading to social isolation. Yet, their ability of emotional repair would help to manage the feeling of loneliness amid this pandemic despite the lack of social interaction. It is well documented that emotional repair is closely related to the ability to control intrusive and ruminative thoughts that would occur with stressful situations (Salovey et al., 1995). A study by Fischer and Phillips (1982) reported that people with a small social network still had a lower level of loneliness. Our research finding is consistent with the literature in that although all the participants in this study experienced loneliness (ranging from low to high), those with a higher level of emotional repair ability showed a lower level of loneliness. Therefore, it can be argued that social isolation (or physical distancing) does not always lead people with visual impairment to a feeling of loneliness (i.e., perceived loneliness).

This study found individual differences in emotional intelligence and loneliness across the participants with different sociodemographic backgrounds. For example, those with no exercise are likely to experience that the more they pay attention to emotions, the more they feel lonely. There is evidence that an individual with lack of physical exercise is more vulnerable to loneliness as the study by Hawkley, Thisted, & Cacioppo (2009) found a significant association between loneliness and physical activity, which was mediated by emotion regulation, but not by social factors (e.g., social network size, marital status, contact with close ties, and group membership). As people with visual impairment are less likely to have opportunities to engage in physical exercise (Jaarsma, Dekker, Koopmans, Dijkstra, & Geertzen, 2014), they are more likely to result in developing a feeling of loneliness, which could be worsened due to the COVID-19 public health interventions such as stay-at-home orders and social distancing practice.

Other individual differences were also found among the participants grouped by gender and race/ethnicity. Female participants with visual impairment were more likely to feel lonely compared to male counterparts. A recent research (Etheridge & Spantig, 2020) reported that over a third of women (34%) indicated they felt lonely sometimes amid the COVID-19 pandemic and 11% felt lonely often; however, these numbers are lower in men – that is, 23% were lonely sometimes and only 6% were lonely often. Etheridge and Spantig (2020) argued that compared to men, women tend to have a larger size of social networks and meet close friends, but the pandemic prohibits them from social interactions with close friends, eventually leading to higher levels of loneliness. African American participants with visual impairment were more likely than European American participants with visual impairment to show lower emotional attention and repair amid the COVID-19 pandemic. Van Rooy, Alonso, & Viswesvaran (2005) found no statistically significant difference in emotional intelligence between the two race/ethnicity groups (i.e., Caucasians and African Americans/Black), which is prior to the COVID-19 pandemic. Thus, it may hypothetically be argued that pandemic-related situations lead to significant difference between the two race/ethnicity groups. As African American participants with visual impairment resulted in lower emotional attention and repair, there is a concern that African Americans with visual impairment would be more vulnerable to emotional challenges during this pandemic period. There is an immediate need for adequate coping strategies to help them recover from a strong emotion and/or maintain a good emotional well-being. The individual difference between European Americans and African Americans with visual impairment should be further studied to identify what other factors led the two groups to the individual difference in emotional intelligence. For example, 40% of participants in this study declined to answer the question about their household income. As there is evidence that emotion intelligence is closely related to family factors (e.g., subjective perception of family financial status, psychological climate in the family, and strength of subject relations with family members) (Lekaviciene & Antiniene, 2016; Rauf, Tarmidi, Omar, Yaaziz, & Zubir, 2013; Shukla & Srivastava, 2016), the group of participants who did not inform about their household income would probably lead to different research results. Future research should consider more various factors to address the knowledge gap.

In addition to loneliness, the participants with visual impairment experienced other emotions. Recent studies have also reported that general populations have experienced a range of emotions affected by different factors during the COVID-19 pandemic – for example, uncertainty, loneliness, health-related concerns, job security–related stress, work–family conflict, and discrimination (Anderson, Heesterbeek, Klinkenberg, & Hollingsworth, 2020; Blustein et al., 2020; Qiu et al., 2020; Restubog, Ocampo, & Wang, 2020; Rudolph et al., 2020; Van Bavel et al., 2020). In contrast to the general populations, the negative emotions of the participants with visual impairment were additionally affected by the poor accessibility of technology and the loss of the independence due to the pandemic. The participants in this study felt left out when they encountered poor accessibility to information and communication technologies, and felt that no one is nearby in person and online.

The participants also shared their coping strategies, for example, avoid, alter, accept, or adapt, which would contribute to their emotional attention, clarity, and repair during the COVID-19 pandemic. Zacher and Rudolph (2020) explored how general populations coped with emotional distress during the early stages of the COVID-19 pandemic and found that positive affect was related to appraisals of controllable-by-self and active coping while negative affect was related to denial and self-blame. Thus, the coping strategies used by the participants with visual impairment in this study can be considered as the controllable-by-self appraisals and active coping, contributing to positive affect. It would be great if there is a systematical and accessible means to inform populations with visual impairment about the positive affect–related coping strategies to overcome emotional challenges amid the COVID-19 pandemic.

## Limitations

This research study may have limitations that affected the results. Loneliness and emotional intelligence were measured using questionnaires that were administered via self-report. The participants may answer to describe himself or herself in the best possible light, resulting in distorted responses to the self-report measurements. However, we informed the participants that their responses would be confidential and any personally identifiable information would be removed or replaced with a random participant number. We examined the correlation between variables so that the direction of causality was not explored. Approximately 40% of the participants declined to answer the question of household income, probably affecting the data analysis results of correlation between loneliness and emotional intelligence among participants grouped by the sociodemographic factor, household income. Our future research will be conducted to address those gaps.

## Conclusion

This study revealed that people with visual impairment are vulnerable to the feeling of loneliness amid the COVID-19 pandemic. Those with different degrees of loneliness are likely to show different degrees to which they pay attention to emotions, clarify emotions, and regulate emotions, which are likely to be affected by various sociodemographic factors. As it is well documented (Morahan-Martin & Schumacher, 2003) that the increased use of the Internet is often observed in lonely people who manage their loneliness, future research will examine the degree to which an Internet-based intervention contributes to managing emotional challenges among people with visual impairment. In particular, as there is a report (Fischer & Phillips, 1982) that despite a small-sized social network people tend to show a lower level of loneliness, future research will investigate how efficiently Internet-based interventions can help people with visual impairment to manage their loneliness via a small-sized social network. Our future study will also consider more various protentional factors (e.g., household incomes and other sociodemographic factors) to investigate the relationships with emotional intelligence and loneliness. Given the findings that people with visual impairment have been struggling with emotional challenges today, adequate coping strategies should be offered to those with visual impairment.
